# A riddle wrapped in an enigma: acute kidney injury in a girl with Crohn’s disease: Answers

**DOI:** 10.1007/s00467-020-04538-y

**Published:** 2020-03-30

**Authors:** Lilach C. Regev, Antonia H.M. Bouts, Jaap W. Groothoff, Joanna A.E. van Wijk, Michiel van Wijk, Paul van der Valk, Arend Bökenkamp

**Affiliations:** 1 Edmond and Lily Safra Children’s Hospital, Sheba Medical Center, Ramat-Gan, Israel; 2 Department of Pediatric Nephrology, Emma Children’s Hospital, Amsterdam University Medical Centers, Amsterdam, The Netherlands; 3 Department of Pediatric Gastroenterology and Nutrition, Emma Children’s Hospital, Amsterdam University Medical Centers, Amsterdam, The Netherlands; 4 Department of Pathology, location VU medical center, Amsterdam University Medical Centers, Amsterdam, The Netherlands

## Answers

1. Does this patient have AKI?

Despite the normal BUN, the patient does have acute kidney injury (AKI). The two most commonly used definitions of AKI in children are the KDIGO and pRIFLE definitions. Both use serum creatinine and urine output to classify AKI, while urea is not included in either classification system. The KDIGO definition defines acute kidney injury as an increase in creatinine of ≥ 0.3 mg/dL in 48 h or ≥ 1.5 times the baseline value within the last 7 days, or a decrease in urine output to less than 0.5 mL/kg/h for at least 6 h [1]. The pRIFLE classification uses three levels of acute injury (risk, injury, and failure) starting with a decrease in eGFR by at least 25% or decreased urine output below 0.5 mL/kg per hour for 8 h [2]. In our case, an eGFR of 26 mL/min per 1.73 m^2^ and decreased urine output of < 0.3 mL/kg per hour for 24 h indicate AKI according to both classifications.

2. How do you explain the discrepancy between high creatinine and normal BUN?

Urea—an end product of protein metabolism—is formed in the hepatic urea cycle to detoxify ammonia. Extrarenal factors that may cause elevated urea levels include excessive protein ingestion, gastrointestinal hemorrhage, catabolic states, and glucocorticoid therapy, whereas very low protein ingestion (e.g., kwashiorkor and marasmus), severe liver disease, or a urea-cycle defect cause low urea concentrations [3]. Low urea concentrations have also been reported in anorexia nervosa [4].

Following glomerular filtration, there is extensive tubular handling of urea. Urea transport in the thin descending limb of Henle and the medullary collecting duct is increased by antidiuretic hormone leading to decreased urea excretion in high anti-diuresis scenarios [3]. Therefore, the fractional excretion of urea (FE_Urea_) may help differentiate between pre-renal and intrinsic renal failure: FE_Urea_ < 35% suggests hypovolemia while FE_Urea_ > 50% suggests acute tubular necrosis (ATN) [5].

In view of profound weight loss over the last month and the absence of polyuria, low protein intake and malnutrition due to Crohn’s disease are the most likely explanation for the discrepancy between creatinine and urea in our patient. The resulting low osmolar load is also reflected by the remarkably low urine osmolality of 118 msom/kg.

3. What is the differential diagnosis of her renal condition?

The differential diagnosis includes pre-renal and intrinsic renal failure. Post-renal AKI is unlikely given the normal ultrasound. The low FE_Na_ might suggest pre-renal AKI, still a weight gain of 1.5 kg prior to admission, low urine osmolality, proteinuria, and a urine sediment containing leukocytes and epithelial cells suggest an intrinsic renal cause. Of note, an FE_Urea_ of 43% is too high to suggest hypovolemia, but not high enough for the diagnosis of intrinsic AKI. This probably reflects the low urea production, which resulted in low serum and urine urea concentrations.

4. What is the most likely type of renal injury in this case?

In view of the extensive list of drugs administered in the weeks prior to admission, either direct drug toxicity (“ATN”) or a tubulointerstitial nephritis (TIN) is the most likely. Still, renal disease has also been linked to inflammatory bowel disease (IBD), major manifestations being nephrolithiasis, TIN, glomerulonephritis, in particular IgA nephropathy, and amyloidosis [6]. While nephrolithiasis was excluded by ultrasound, the rapid deterioration of kidney function and the absence of hematuria and significant proteinuria argue against IgA nephropathy. The history of Crohn’s disease was too short to consider amyloidosis as a differential diagnosis.

## Discussion

We present a patient with AKI shortly after the diagnosis of complex Crohn’s disease who had been treated with steroids, azathioprine, infliximab, and multiple antibiotics in the weeks before presentation. Due to the low FE_Na_, she was given a fluid challenge with normal saline on presentation, yet this did not improve urine output nor kidney function. Therefore, drug toxicity or a TIN was deemed likely and all medication was withheld.

*Tubulointerstitial nephritis* is an immune-mediated tubulointerstitial inflammation with sparing of the glomeruli and vessels. Patients commonly present with nonspecific symptoms including fever, malaise, flank pain, anorexia, weight loss, myalgia, arthralgia, and skin rash. Renal symptoms include polyuria due to impaired renal concentrating capacity. Blood tests may show anemia, eosinophilia, elevated C-reactive protein, and erythrocyte sedimentation rate. Urinalysis is characterized by sterile pyuria with WBC casts, hematuria, and non-nephrotic range proteinuria of tubular origin [7, 8]. Granular casts and eosinophiluria may also be found. Renal ultrasonography often demonstrates nephromegaly with increased echogenicity [8].

Renal biopsy demonstrates infiltration of the renal interstitium, predominantly by lymphocytes and monocytes accompanied by smaller numbers of eosinophils, plasma cells, neutrophils, histiocytes, and mast cells. Other findings are interstitial edema, and tubulitis represented by tubular inflammatory infiltration and tubular degenerative changes, while blood vessels and glomeruli are typically spared. Interstitial eosinophils suggest drug-induced TIN, whereas the presence of neutrophils favors bacterial infection [8]. Granulomatous interstitial nephritis may be seen with exposure to beta-lactam antibiotics, infections such as tuberculosis and granulomatous inflammatory diseases such as sarcoidosis [9].

TIN is usually caused by drugs, infections, or autoimmune diseases such as TINU (tubulointerstitial nephritis with uveitis), SLE, or sarcoidosis [8]. A large number of drugs have been reported to cause TIN, the most commonly implicated being NSAIDs and beta-lactamase antibiotics. Renal symptoms develop on average 10 days after first-time exposure, with 80% of patients presenting the symptoms within 3 weeks after exposure [10]. In the present case, ciprofloxacin, NSAIDs, and azathioprine were considered the most likely culprits. Recently, infliximab has also been reported to cause TIN [11, 12] and there has been a single report of tramadol as a cause of granulomatous interstitial nephritis [13].

TIN is seen frequently in patients with IBD [6] as it is linked to the use of mesalamine, but this was not prescribed in our patient as it is used in ulcerative colitis rather than Crohn’s disease [10]. TIN has also been observed in IBD patients without previous medical treatment [14].

Discontinuing exposure to the drug responsible for TIN is the most important intervention and should be done as soon as possible. Treatment with corticosteroids is controversial as it has been shown to hasten recovery but not to decrease the overall risk of chronic renal failure. Still, randomized controlled trials are lacking and steroid treatment is warranted in immune-mediated TIN such as TINU or SLE [15].

*Acute tubular necrosis* (ATN) may result from *direct* tubular epithelial injury by drugs or more commonly by ischemic injury or sepsis. Patients usually present with a recent history of hypoperfusion or a possible toxic exposure. Urinalysis classically reveals muddy brown granular casts, epithelial cells without red blood cells or white blood cells [16]. As in TIN, urine sodium is usually increased (> 40–50 mEq/L) [16, 17] with FE_Na_ above 2% [16] and low urine osmolality due to loss of concentrating ability [17]. In contrast to TIN, patients usually present with oliguria [16].

Histological examination shows ischemic changes ranging from swelling of tubular cells to focal tubular epithelial necrosis and apoptosis with desquamation of cells into the tubular lumen, dilated proximal tubules with loss or thinning of brush border, interstitial edema, and eosinophilic hyaline casts of Tamm-Horsfall protein [16].

Drugs commonly associated with ATN include aminoglycosides, vancomycin, radiocontrast media, calcineurin inhibitors, cisplatin, NSAIDs [16], synthetic cannabinoids [18], and ciprofloxacin [19, 20].

The mainstay of treatment of ATN is preventive and supportive [1, 16]. Prognosis is variable and depends on the underlying condition, presence of oliguria, need for renal replacement therapy, and age [16].

Our patient did not represent the classic clinical picture of either of the two options—leukocyturia, leukocytosis, and flank pain suggested TIN, while oliguria, weight gain, and epithelial cells in the urinary sediment suggested ATN. Furthermore, when reviewing the patient’s exposures, we found multiple medications (i.e., ciprofloxacin, azathioprine, infliximab, augmentin, and ibuprofen) along with IBD itself that could cause TIN, while others are known causes of ATN (gentamicin, ibuprofen, synthetic cannabinoids, and again ciprofloxacin). Our patient used a minimal amount of medicinal cannabinoid extract, which—in contrast to synthetic cannabinoids—has not been linked to ATN [21], so this was less likely.

**Fig. 1:**
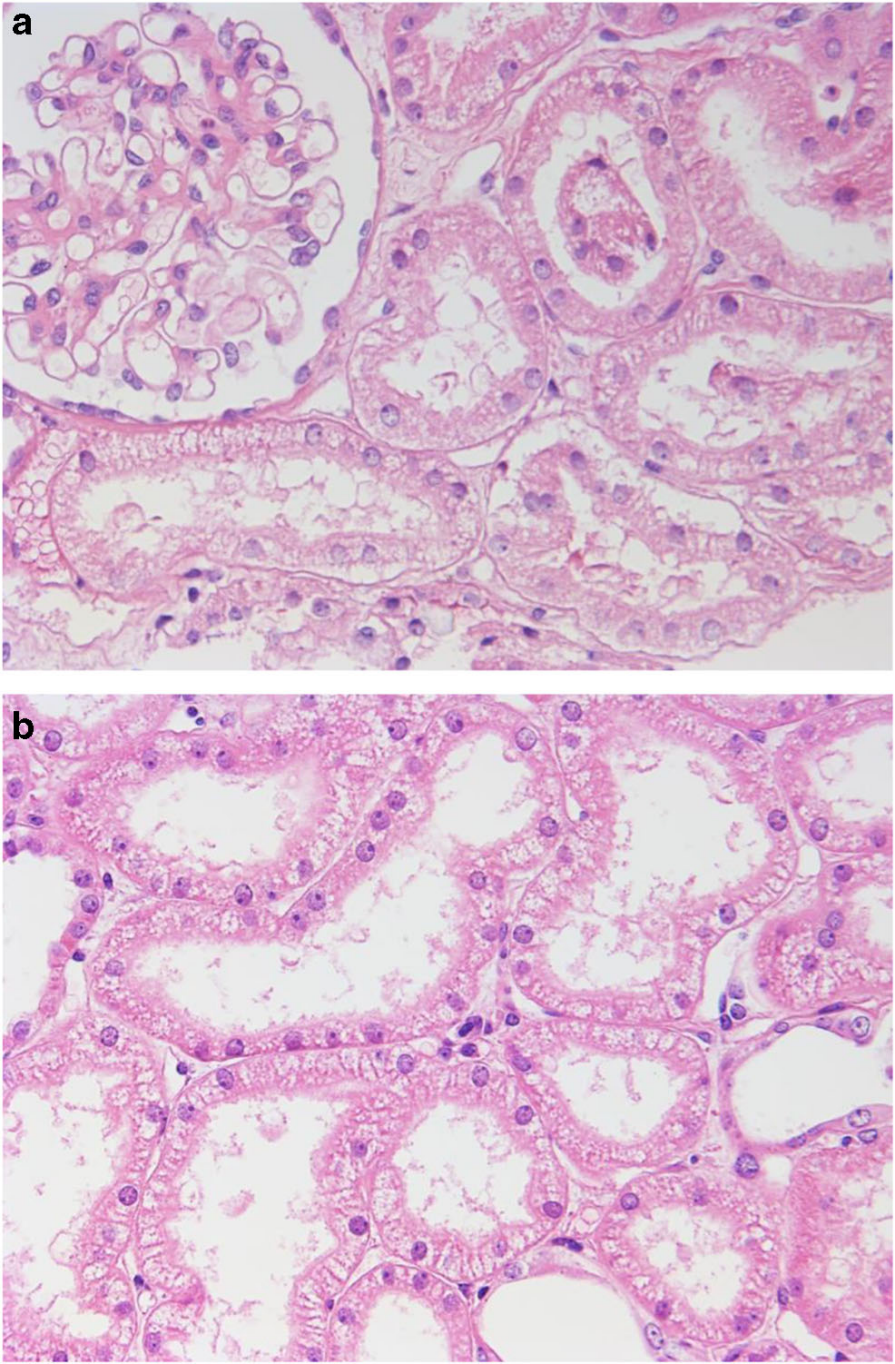
Renal biopsy, H&E staining. **a** Normal glomerular histology, hydropic tubular epithelial cells. **b** Tubular epithelial cells with hydropic changes and vacuoles

Despite cessation of all medication and prednisone, starting on day 3, we observed a rapidly progressive rise in creatinine and urea which prompted a renal biopsy taken on day 5. Histological examination showed hydropic tubular epithelial cells with irregular cellular vacuoles consistent with the diagnosis of vacuolar ATN (Fig. 1 a and b). The absence of an inflammatory infiltrate ruled out TIN; glomerular histology was normal.

There have been several reports on ATN caused by ciprofloxacin [19, 20, 22–24]. Ciprofloxacin-induced ATN has been described most frequently in cases of drug overdose [20], the ciprofloxacin dose of 36 mg/kg/day is higher than commonly used (30 mg/kg/day) but commonly prescribed in patients with cystic fibrosis [19]. Hydropic or vacuolar ATN has been described following osmotic challenge like radiocontrast media, sucrose-containing intravenous immunoglobulin preparations, following ethylene-glycol ingestion and in calcineurin inhibitor toxicity. Gerritsen et al. described a case of ciprofloxacin-induced nephrotoxicity with epithelial vacuoles seen in the distal tubule on electron microscopy resembling the vacuoles seen in our case [19]. Their patient had received normal doses of ciprofloxacin.

The patient was dialyzed twice before renal function started to improve. On follow-up 4 weeks after admission, renal function had normalized. Infliximab treatment was resumed 2 weeks after the last dialysis session without adverse events.

## Conclusion

Differentiating TIN from ATN can be challenging, in particular in cases with multiple potential causes. Kidney biopsy may be indicated if corticosteroid treatment is considered in progressive kidney failure to diagnose presumptive TIN. Our patient had received high-dose ciprofloxacin treatment for 14 days until admission, making ciprofloxacin the most likely culprit causing ATN while the other potentially nephrotoxic substances had been used much earlier and for very short duration.
